# Psychological Factors Including Demographic Features, Mental Illnesses, and Personality Disorders as Predictors in Internet Addiction Disorder 

**Published:** 2018-04

**Authors:** Malihe Farahani, Seyyed Salman Alavi, Mahmood Mirzamani Bafghi, Sudeh Esmaili Alamuti, Zohreh Taghavi, Mohammadreza Mohammadi

**Affiliations:** 1 West Tehran Branch-Azad University, Department of Psychology, Tehran, Iran.; 2 Psychiatry and Psychology Research Center, Tehran University of Medical Sciences, Tehran, Iran.; 3 Department of Psychology, College of Education and Psychology, Islamshahr Branch, Islamic Azad University, Islamshahr, Iran.; 4 Department of Psychology, Allame Tabataba’i University, Tehran, Iran.

**Keywords:** *PsychologicalFactors*, *Internet Addiction Disorder*, *Mental Illnesses*, *Personality Disorders*

## Abstract

**Objective:** Problematic internet use is an important social problem among adolescents and has become a global health issue. This study identified predictors and patterns of problematic internet use among adult students.

**Method** **:** In this study, 401 students were recruited using stratified sampling technique. Participants were selected among students from 4 universities in Tehran and Karaj, Iran, during 2016 and 2017. Internet Addiction Test (IAT), Millon Clinical Multiaxial Inventory - Third Edition (MCMI-III), Structured Clinical Interview for DSM (SCID-I), and semi-structured interview were used to diagnose internet addiction. Then, the association between main psychiatric disorders and internet addiction was surveyed. Data were analyzed using SPSS18 software by performing descriptive statistics and multiple logistic regression analysis methods. P- Values less than 0.05 were considered statistically significant.

**Results:** After controlling the demographic variables, it was found that narcissistic personality disorder, obsessive- compulsive personality disorder, anxiety, bipolar disorders, depression, and phobia could increase the odds ratio (OR) of internet addiction by 2.1, 1.1, 2.6, 1.1, 2.2 and 2.5-folds, respectively (p-value<0.05), however, other psychiatric or personality disorders did not have a significant effect on the equation.

**Conclusion: **The findings of this study revealed that some mental disorders affect internet addiction. Considering the sensitivity and importance of the cyberspace, it is necessary to evaluate mental disorders that correlate with internet addiction.

The term addiction was originally associated with drug ingestion and alcohol consumption. However, there is an ongoing change in the field that opens up a number of behaviors as potentially addictive (1, 2). Recently, there has been increasing focus on technological addiction in light of the fact that behavioral addictions have begun to be more widely studied. Technological addiction shares some characteristics and shows similarities with several other established types of behavioral addiction and could be seen as a subcategory of behavioral addiction (1, 3, 4). Internet addiction (IA) is not currently categorized by the DSM-5 as a formal psychiatric disorder, but it is listed in Section 3 in a modified form, Internet Gaming Disorder as a disorder requiring further research (5). Individuals with internet addiction typically spend 40 to 80 hours per week online (6), spend more time on the internet for nonessential use (i.e., pleasure, hobby, or personal use), and report a variety of problems associated with their internet use (7, 8). 

A growing volume of research done in this field suggests that internet addiction disorder is a psycho-social disorder.

The characteristics of this disorder include tolerance, withdrawal symptoms, emotional disorders, and fragmentation of social relations (1). Internet addiction (IA) is an actual addiction like substance addiction and other forms of dependence. It is a pervasive, chronic, and recurrent phenomenon correlated with serious family, social, physical, financial, and psychological injuries. The addicted persons experience a significant drop in interpersonal and social functions (9). Many studies have reported associations among internet addiction, psychiatric symptoms, and depression among adolescents. Many studies found that excessive internet use is also associated with the presence of psychological problems, such as academic, family, and educational failure (10-13). University counselors had reported similar problems. For example, in a study on 224 students at University of Isfahan, it was found that 14% of the participants met the criteria of internet addiction (14). Research shows that depression, hostility, social phobia, anxiety, and symptoms of ADHD are seen as comorbid conditions to problematic internet use.

 Yen et al. reported that adolescents with internet addiction had higher symptoms of attention deficit hyperactivity disorder (ADHD), social phobia, depression, and hostility. Higher ADHD symptoms, depression, and hostility were correlated with internet addiction in male adolescents, and only depression and higher ADHD symptoms were associated with internet addiction in female students (15). In another study, Ko et al. found that aggression and attention deficit were the highest predictors of internet addiction in both genders (boys and girls) among adolescents (16). Furthermore, studies by Christakis et al. and Catriona reported that persons who suffer from internet addiction are more depressed than non-addicted ones. Researches showed higher rate of internet addiction among males than females and in adolescents than in adults. There was a significant correlation between depression levels and internet addiction disorder (17, 18). In another study conducted by Alavi et al. on 250 students, it was that there were differences between the internet addicted group and normal users in the scores of obsessive-compulsive disorder, interpersonal sensitivity, somatization, anxiety, depression, aggression, paranoid, and phobic anxiety (19).

Despite the fact that more and more researches have reported the effect of internet addiction on psychological disorders, such as anxiety and depression, very few studies have focused on the impact of compulsive internet use on psychiatric symptoms based on psychiatric interview. Also, past studies have achieved contradictory results, and the observed findings are quite limited. Furthermore, published studies on personality disorders and their correlation with internet addiction are rare (20). Montag et al. (2017) reported that the risk factors of internet addiction can be categorized as psychological, social, or biological factors (21).

This study was conducted to evaluate the specific hypothesis that mental disorders and personality disorders can predict internet addiction. The present study was conducted to determine the psychological factors that influence internet addiction. In addition, the presence of mental disorders were compared in 2 groups of students with internet addiction, and those without internet addiction. Iran is not immune from negative effects of internet addiction. Thus, designing appropriate models to identify factors affecting internet addiction in Iran is deemed necessary.

## Materials and Methods

***Study Design***

In this cross-sectional study, 401 students (male/female) were selected from 4 universities in Tehran and Karaj, Iran. Inclusion criteria were age 18 to 30 years and using the internet for a minimum of 1 hour daily for past two years. Students with history of severe psychopathology, which could prevent active participation in the study and interview (according to the psychologist’s diagnosis), and those who were not willing to participate were excluded from the study.

***Tools***

**1) Demographic Questionnaire:** Background data sheet was designed by the investigator to record socio-demographic details, which covered gender, age, socioeconomic status (low, middle, high), marital status, education level, internet use, age at which the person started using it, type of information technology used, reason to start using the internet, frequency of use, sites accessed, duration of use, having a smart phone, availability of internet at home, purpose of using the internet or other technology devices, any history of attempt to reduce the usage of internet, etc.

**2) Internet Addiction Test (IAT):** IAT is a 20- item self-report questionnaire based on the DSM-IV diagnostic criteria for pathological gambling and alcoholism. It includes questions that reflect typical behaviors of addiction. IAT comprises the following components: obsessive behavior related to internet or chatting, withdrawal symptoms, tolerance, slump in school performance, negligence of family and school life, difficulties in personal relationship, behavioral problems, health trouble, and emotional problems. The severity of addiction was then classified according to the suggested 20-49, 50-79, and 80-100 scores as normal, moderate, and severe, respectively. In the present study, we used the Persian version of IAT, which had a Cronbach's alpha reliability of 0.89, and the p-value of test-retest was 0.68 after 2 weeks. Minimum score on this scale is 20 and maximum is 100. The scale showed moderate to good internal consistency and was validated by its personal and general internet usage (22, 23). 

**3) Semi-structured interview based on DSM-IV-TR:** criteria for impulse control disorder not otherwise specified (ICD-NOS) was performed by a clinical psychologist trained for the diagnosis of impulse control disorder (ICD) in general and internet addiction disorder in particular.

**4) Structured Clinical Interview for DSM (SCID-I):** Psychologists conducted the clinical interview to diagnose any mental disorder in participants. The interviews were done by clinical psychologists who had been educated in the field of diagnosis and treatment of behavioral disorders. The interviews were performed in a quiet place at students’ convenience. The SCID is a diagnostic test used to determine major mental disorders (SCID-I). Due to its high level of reliability and good validity, this standardized semi-structured clinical interview for DSM-IV (SCID-I) is a valid structured interview and the gold standard for assessing DSM-IV Axis I disorders. It was assessed by independent, trained, and supervised clinicians, and it is recommended as the gold standard tool for diagnosing mental disorders (24). In another study, Duarte-Guerra et al. (2015) reported the inter-rater agreement for SCID-I lifetime disorders to be as kappa coefficient of 0.81 in a random sample of 15 patients (25). This interview evaluated 16 main psychology and psychiatric disorders and other psychiatric disorders including psychiatric problems, depression, mania, hypomania, substance abuse and addiction, psychosis, anxiety disorders, antisocial personality, somatization disorders, suicidal thoughts, posttraumatic stress disorders (PTSD), dissociative disorders, epilepsy disorder, Alzheimer, and mental retardation.

**5) Millon Clinical Multiaxial Inventory - Third Edition (MCMI-III):** Millon Clinical Multiaxial Inventory - Third Edition (MCMI-III) is the most recent edition of the Millon Clinical Multiaxial Inventory. The MCMI is an assessment tool to provide information on personality traits, personality disorders, and psychopathology including specific psychological and psychiatric disorders determined in the DSM-IV. It is designed for adults (18 and over) with at least a fifth grade reading level. The MCMI was standardized and developed specifically on clinical populations (ie, patients in clinical settings or persons with existing mental health problems) (26). The MCMI-III was presented in 1994 and reflected revisions made in the DSM-IV. This version omitted specific personality scales and added new scales for PTSD and depression, bringing the total number of scales to 14 personality scales, 10 clinical syndrome scales, and 5 correction scales. The third edition form consists of 175 false- true items that take approximately 20 to 25 minutes to complete. This inventory is almost self-report. Results of Blais et al. indicated that the MCMI-III Avoidant scale is reliable (r = 0.89) and it was found to demonstrate appropriate divergent and convergent validity with other self-administrating measures. The MCMI-III Anxiety scale also showed satisfactory reliability (r = 0.78) (27). 

***Procedure***

Participants were taken from 4 universities in Tehran and Karaj. The objectives and process of the study were explained to the students and informed consent was obtained. The socio-demographic information was filled as per the information given by the samples, and the internet addiction questionnaire, MCMI, SCID-I, and screening interview for psychiatric disorders were administered in individual setting. The semi-structured interviews were performed by the same 2 specially trained psychologists.

*Statistical Analysis*

Descriptive statistics were used to demonstrate demographic data. Chi-square was used to assess the significance of the associations among the variables. The mental disorders that contribute to internet addiction were determined using logistic regression analysis. P-values less than 0.05 were considered as statistically significant.

To analyze the data, all variables including personality disorders, mental illnesses, and demographic characteristics that were collected through questionnaires and were likely to have an impact on internet addiction were identified. Logistic regression analysis was conducted for each variable in the univariable analysis and crude OR was also estimated. In the next step, variables with p- values <0.2 were selected and entered into the logistic regression analysis. Then, those variables that had a significant role in the model with p- values<0.05 were interpreted with adjusted OR*.*

## Results

The mean age of the participants was 25.3 with the standard deviation of 4.6. Age distribution was 18 to 30 years. The participants included 127 males (31.7%) and 274 females (68.3%). Of the participants, 261 were single (65.1 %) and 140 married (34.9%). There were no differences between the participants in their demographic information (Table 1).

As can be seen in Table 2, The results of this study revealed that age did not increase the odds of internet addiction occurrence. In addition, according to our results, male students had a tendency to use the internet more frequently than females. The odds of internet addiction in males were about 50% more than in females. We found that the odds of internet addiction in single individuals were about 1.2 times more than the married. The results revealed that among the personality disorders, narcissistic personality disorder was associated with a 2.1-fold increase in odds of internet addiction and that obsessive compulsive personality disorder increased the chance of internet addiction (OR = 1.1) (negative effect). However, other personality disorders did not seem to have a significant effect on the odds of internet addiction.

The findings revealed that anxiety increased the odds of internet addiction by 2.6-fold. Furthermore, with regards to mood disorders, particularly bipolar disorder, the results showed that this disorder increased the odds of internet addiction by 1.1-fold. 

Depression also increased the odds of internet addiction by 2.2-fold (negative effect). Based on the multiple logistic regression interpretations (multivariable analysis), history of substance abuse had an impact on the probability of internet addiction; moreover, among other mental disorders, social phobia also increased the odds of internet addiction occurrence by 2.5-fold.

Our results showed that some mental disorders, such as eating disorders (including bulimia nervosa or anorexia nervosa), posttraumatic stress disorder (PTSD), histrionic personality disorders, and other socio-demographic variables (including time usage of internet per day) were not significant parameters in the equation. Moreover, none of the participants mentioned any mental disorders (including psychotic symptoms (delusion or hallucination)), history of epilepsy attack, hypomania, fugue, or other mental problems, so we did not enter any of these disorders into the equation.

## Discussion

The purpose of this research was to analyze the internet addiction among a sample of students and determine the associations between psychiatric disorders and excessive internet use.

The results revealed that age of the students was not a predictor of internet addiction. Asiri et al. reported significant correlations between internet addiction and age (28).Fu et al. found that younger individuals used internet 3 times more than the older (29), and another study reported a significantly higher rate of internet addiction in males than in females and a higher rate of internet addiction in adolescents than in adults (18).

However, lee and Stapinski, similar to our results, could not report a significant association between age and internet addiction (30). The incompatibility between our findings and those of previous studies could be due to different populations, cultural conditions, diagnostic tools, and the effect of other variables, such as gender, environmental factors, and history of internet. In mentioned studies, the age range of participants varied, whereas in our study the students were selected from limited age range (18-30 years), which could have affected the results.

**Table 1:** Summarizes Some Socio-Demographic Information of the Participants Based on Their Diagnosis of Internet Addiction

**Demographic Properties**		**Diagnosis of Internet Addiction**		
		**Yes**	**No**	**P-value**
University	Khajeh Nasir Toosi University of Technology	17(20.5%)	64(20.1%)	0.145
	Tehran University	10(12%)	32(10.1%)	
	Tehran University of Medical Sciences	3(3.6%)	7(2.2%)	
	Islamic Azad University of Karaj branch	53(16.7%)	215(67.6%)	
Sex	Female	55(66.3%)	219(68.9%)	0.020
	Male	28(33.7%)	99(31.1%)	
Education	Undergraduate & Bachelor	55(66.3)	119(68.9%)	0.158
	MSc or PhD	28(43.7%)	99(31.1%)	
Marital status	Single	46(55.4%)	215(67.6%)	0.948
	Married	37(44.6%)	103(32.4)	
Age (year)	Mean±SD	25.4±4.5	25.3±4.6	0.81

**Table 2:** Summary of the Effect Size of the Relationship Between all Psychiatric Symptoms Based on OR (adjusted)

**Variables**	**Univariable Analysis**			**Multivariable Analysis**		
	**OR(crude)**	**CI (95%)**	**P-value**	**OR(adjusted)**	**CI (95%)**	**P-value**
Age	1.05	1.05-1.1	0.02	1.02	0.96-1.1	0.41
Gender (male VS female)	1.4	0.92-1.2	0.11	1.5*	1.3-2.4	0.05
Marital status (single vs. married	1.4	0.95-2.1	0.08	1.2*	1.4-1.8	0.05
Duration of using the Internet	1.2	0.91-1.6	0.17	1.1	0.8-1.5	0.53
Narcissistic personality disorder	1.34	0.98-1.8	.063	2.1*	1.3-3.4	0.002
Antisocial personality disorder	0.26	0.42-1.7	0.16	0.70	0.14-3.3	0.65
Compulsive personality disorder	1.1	0.71-1.51	0.14	1.1*	1.5-2.4	0.04
Anxiety	7.9	0-0.7	0.001	2.6*	1.3-5.5	0.05
Bipolar disorders	1.2	0.65-2.3	0.20	1.1*	1.6-6.3	0.040
Depression	3.9	078-19.2	0.09	2.2*	3.8-12.6	0.03
Substance abuse	4.2	1.2-4.3	0.05	1.4*	3.1-4.6	0.05
Bulimia	1.5	0.67-3.2	0.19	1.4	0.6-3.4	0.4
Social phobia	2.7	0.52-14.1	0.2	2.5*	4.6-13.5	0.02

According to our findings, single persons tend to use internet more frequently than married ones. The odds of internet addiction in single persons were about 20% more than married ones. Similarly, other studies reported that compulsive internet use was correlated with marital status, gender, and educational level (28, 31). Chou et al. reported that the risk of excessive internet use was higher in single participants. Being single and unstable family relationships were the risk factors for internet addiction (32). Also, another study reported that unmarried male adolescents had a higher tendency toward internet use and were at more risk of internet addiction (33, 34).

It can be inferred that since the single, compared to the married, feel lonelier and this drives them more towards virtual spaces, causing dependence.

The results revealed that among the personality disorders, compulsive personality disorder was 1.1-fold more likely to increase internet addiction probability. These results are compatible with other studies and support previous findings. Ge, Se, and Zhang reported that the score of compulsive internet use and its related dimensions can serve as indicators of neuroticism, psychoticism, and mental health (35).

In this study, results revealed that among the personality disorders, narcissistic personality disorder was 2.1-fold more likely to enhance internet addiction probability. 

Similarly, in one study 21 individuals who reported pathological computer use were assessed and it was found that 52% of participants had one personality disorder with the highest frequencies in narcissistic, borderline, and antisocial personality disorders (36). Bernardi and Pallanti (2009) assessed 15 individuals who suffered from internet addiction, and the findings indicated that among those with internet addiction, 14% had symptoms of borderline, 7% had symptoms of avoidant, and 7% had symptoms of obsessive- compulsive personality disorder (37). Floros et al. (2014) reported that 38% of students who presented with any internet addiction symptoms had personality disorder (38). 

In another study, results revealed an association between IA and personality disorders using stratifying analyses. This study found higher rates of personality disorders in the internet addicted group (27.4%) than in a group without internet addiction (13.9%). Furthermore, it was indicated that the IA group had higher symptoms of each personality disorders, such as dependent, narcissistic, borderline, and avoidant disorders (39).

It has been inferred that individuals with personality disorders (PD) are more likely to develop internet addiction. The virtual environment might be very attractive for persons with personality disorders, and individuals with each personality disorder might escape from their undesirable feelings, such as loneliness, affective instability, grandiosity etc., using social networking sites or chatting, which are the best way to escape from unpleasant feelings. Thus, using the internet is a coping strategy for social or emotional difficulties. 

Overall, the results support the hypotheses about the correlation of internet addiction and personality disorders. Persons with personality disorders (PD), especially with personality disorders associated with low self-esteem and high impulsivity (Cluster C and Cluster B personality disorders) might be at special risk for developing internet addiction (IA). Affected individuals might tend to use online applications in a maladaptive manner to cope with interpersonal and emotional difficulties in the real world. As a consequence, personality disorders might be a perpetuating factor for IA and vice versa. If social difficulties in the real world do not improve, individuals may receive and satisfy social motivation predominantly from the internet, and then, the risk of becoming addicted to the internet may increase and individuals may develop increased impairments in real-life situations.

In addition, our study shed light on the impact of psychological disorders on internet addiction, which means that internet addiction had various comorbidities, such as anxiety or depression. Our results showed that anxiety increased the odds of internet addiction by 2.6-fold. Previous reports demonstrated that anxiety, ADHD, and depression are the 3 psychological disorders that are prevalent in individuals with internet addiction (40). Also, other researchers have found that shyness and social anxiety are significant predictors of internet addiction (15, 16, 41). In another study, Montag and Reuter have cited that an individual suffering from anxiety symptoms may seek companionship in a safe cyberspace (21). Also, Laconi et al. (2018) highlighted the relationships between pathological internet use, time spent online, and psychopathology (42). Nevertheless, in a study, it has been claimed that the relationships of internet addiction with aspects of depression and anxiety need to be further studied (43).

There are a number of possible definitions for the association between anxiety symptoms and internet addiction symptoms in adolescents. The first point we would like to address is stress from weak interpersonal relationships in real- life, which may encourage adolescents to experience a high level of anxiety symptoms, leading to an escape into the virtual world of the internet.

Next, anxiety symptoms may make it difficult for children or adolescents to achieve in the real world; thus, adolescents with anxiety symptoms may seek achievements online via virtual spaces instead of in the real world. 

Our findings revealed that depression increased the risk of internet addiction by 2.2-fold (negative effect). Previous studies have found a significant association between internet addiction (IA) and depression among adolescents (14, 16, 33, 44). Also, Whang et al. reported a significant association between the various level of internet addiction and negative emotions, such as depression, loneliness, and obsessive behavior (45). Ha et al. (2007) found that internet addiction was correlated with depressed mood and obsessive-compulsive behaviors (46). The internet provides the youths who suffer from depression the opportunity to escape from emotional turbulence in the real world. It is rational to hypothesize that individuals who have a depressed mood or feel worthless are more likely to overuse the internet to mitigate their negative mood than their non-affected peers, therefore, symptoms, such as low self-esteem in the real world, sadness, hopelessness, etc. may increase the odds of internet addiction.

These results suggest that internet use may provide space for persons to escape from stress or sadness in the real world. These individuals tend to become more vulnerable to maladaptive behaviors and interpersonal dangers than others. However, the causal correlation between depression or anxiety and internet addiction needs to be further assessed in longitudinal and prospective studies. 

With regards to mood disorders, particularly bipolar disorder, the results showed that this disorder increased the odds of internet addiction by 1.1-fold. In an investigation, Shapira et al. (2000) reported that 55% of persons suffering from internet addiction met criteria for bipolar disorder (7). Bernardi and Pallanti reported that 13% of patients being treated for internet addiction suffered from hypomania (37), and Ko et al. proposed to describe manic episodes as an exclusion criterion for IA (10). Wolfling et al. (2015) cited that gambling behavior during manic episode has been defined as an exclusion criterion. Moreover, considering that there are similarities between gambling disorder and internet addiction, systematic investigations regarding frequency of bipolar spectrum disorder (BSD) in internet addiction are needed to better define diagnostic boundaries (47). In a research, a relationship was found between internet addiction and bipolar disorders according to the Mood Disorders Questionnaire (MDQ). Meanwhile, the association between the score of MDQ and time spent online on a day of the weekend was significant. According to their results, bipolar disorders (BD) and internet addiction (IA) show high rates of comorbidity (47).

According to the obtained data, social phobia increased the risk of internet addiction by 2.5-fold (negative effect). Yen et al. reported that the youth with internet addiction suffer from disorders, such as attention deficit hyperactivity disorder (ADHD, social phobia, depression, and hostility (15). The students with high level social phobia scores do not prefer face to face social communication or interactions with other people. Thus, more and more they communicate with other people in the virtual world, and this will lead to addiction to the internet.

On the other hand, despite these findings, some researches did not associate internet addiction with psychiatric disorders such depression, social anxiety, and frustration (14, 48). The inconsistency between our results and those in the above-mentioned studies could be due to different populations and the effect of other main variables such as age, sex, marital status, or the amount of time spent using the internet.

Findings on the effects of mental state on overuse of the internet are inconclusive. However, altogether, the general health of internet addicts is more at risk than that of non- internet addicts.

Thus, it is needed to definite various demographic criteria to increase the comparative ability of the results. Future research should focus on the role that excessive internet use plays in the development of psychiatric disorders, such as obsessive-compulsive, anxiety, or depression. Since it has yet to be determined whether psychological symptoms are the cause or the outcome of internet addiction, researchers need to conduct longitudinal research on the internet and its users.

Study Strengths: This was one of a very few studies to examine psychiatric disorders or personality disorders with clinical interview (SCID). We assessed 400 participants with different main socio-demographic backgrounds such as sex, age, or marital status. Another strength of the study was the diagnostic procedure of internet addiction based on questionnaire and interview. The findings of this study provide essential information that can contribute to diagnosis, prevention, and model designing of internet addiction.

## Limitation

This study had some limitations: First, the analyses were based on a selective sample (students), thus, it is uncertain whether these results can be generalized to other population settings. Second, the cross-sectional method of the study limited the possibility of drawing conclusions regarding causal relationships, and thus findings have to be interpreted with caution.

## Conclusion

The complexity of human behavior comes from various factors, such as cultural, economic, educational, and social, which can affect the mental illnesses and may play an important role in shaping the behavior. The results of the present study provide basic information that might contribute to the diagnosis, prevention, or treatment of internet addiction among students. Considering the mental problems caused by the use of the internet, it is of paramount importance to promote the culture of internet use in the society and families by providing appropriate education. 
